# Medical Obituaries—Dr. McClellan—Dr. Cooke—Dr. Howard

**Published:** 1854-03

**Authors:** 


### MEDICAL OBITUARIES.

Died, at Philadelphia, Jan. 4th, Dr. Samuel McClellan, aged 54 years. He was brother of Dr. George McClellan, the late distinguished surgeon of that city. Both brothers lectured in the Jefferson College from its commencement, in which Dr. Samuel McClellan was formerly Professor of Obstetrics, though he afterwards lectured on the same branch in the Pennsylvania College. He was in the enjoyment of an extensive obstetric practice, and his death is said to have been owing to fatigue from over-exertion. His death was much regretted by his numerous friends, both public and professional.

Died, Dr. John Esten Cooke, on the 30th of October, in the 70th year of his age. Dr. Cooke succeeded Dr. Drake in the Chair of the Theory and Practice in Transylvania University, and was subsequently connected with the Medical Institute of Louisville. He was a voluminous writer. Beside his treatise on Pathology and Therapeutics, he wrote for the American Medical Recorder, the Transylvania Journal of Medicine, and occasionally on theological subjects.

Drs. Drake and Cooke were fellow students in Philadelphia in 1805.

It is with profound sorrow and deep-felt regret, that we are compelled to record the untimely death of R. L. Howard, M. D., Professor of Surgery in the Starling Medical College of Columbus, Ohio.— He died recently, of Pneumonia complicated (says a private letter) with Bright's Kidney. We have known him long and well, and can testify to his sterling qualities as a man, his rare acquirements and success as a physician and surgeon, and his abilities as an instructor. The place made vacant by his death, it will be very difficult to fill, either in the flourishing institution with which he was connected, in the ranks of the profession, or in the community in which he lived and labored.

Dr. Howard was a good surgeon. We do not mean by this, that he was a good manual operator only, but that he understood and practiced this department in its legitimate sense. He sought not display in the use of the knife, but operated only where there was a reasonable prospect of success. In those cases, where there was no promise of success, he was not reckless enough to rush upon the knife, to inflict upon the system torture and violence under which it must speedily and inevitably sink. He took strong ground against this unhuman, barbarous, and unscientific course.

The "Ohio Medical Journal" was first projected and edited by the late lamented Butterfield, and after his death, it fell into the charge of Prof. S. H. Smith. The standing and abilities of these gentlemen made the journal one among the first of the country. It then passed under the care of Prof. Howard, whose talents not only sustained its previous character for usefulness, but it rapidly increased in public favor.

He was also an able teacher and an impressive lecturer. He was a favorite with the class ; and with his distinguished co-laborers an institution has grown up, by their united efforts, which is now a blessing to the State and the profession. Dr. Howard was in the prime of life and in the zenith of his usefulness. He was gentlemanly and kind, and his mind was imbued with those elevated sentiments of high-souled honor which held him aloft among the great and good of the profession. We tender our sympathies to the family and friends, to the faculty, and to the profession of the State, and trust that his example will be felt and followed by its members in coming time.

